# Knockdown of LncRNA CRNDE suppresses proliferation and P-glycoprotein-mediated multidrug resistance in acute myelocytic leukemia through the Wnt/β-catenin pathway

**DOI:** 10.1042/BSR20193450

**Published:** 2020-06-04

**Authors:** Yiqing Kang, Suping Zhang, Weijie Cao, Dingming Wan, Ling Sun

**Affiliations:** 1 Department of Hematology, The First Affiliated Hospital of Zhengzhou University, No. 1 Jianshe East Road, Zhengzhou 450052, P.R. China; 2 Henan Red Cross Blood Center, No. 9, Tongle Road, Zhengzhou 450012, P.R. China

**Keywords:** acute myelocytic leukemia, LncRNA CRNDE, multidrug resistance, P-glycoprotein, Wnt/β-catenin pathway

## Abstract

Mechanisms involved in non-coding RNAs have been implicated in multidrug resistance (MDR) of acute myeloid leukemia (AML). Long non-coding RNA (lncRNAs) colorectal neoplasia differentially expressed (CRNDE) is reported to be involved in the malignant progression in AML. The purpose of the present study is to explore the roles and potential molecular mechanism of CRNDE in the MDR in AML. In our study, we confirmed that the expression of CRNDE was significantly up-regulated in patients with AML, especially in AML patients after adriamycin (ADR)-based chemotherapy. Spearman correlation analysis showed a positive correlation between the levels of CRNDE and MDR1 in AML patients after ADR-based chemotherapy. Moreover, CRNDE was up-regulated in AML cells, especially in ADR-resistant AML cells. Multidrug resistance protein 1 (MDR1)/p-glycoprotein (P-gp) levels were significantly increased in ADR-resistant AML cells, compared with parental AML cells. CRNDE down-regulation inhibited cell proliferation, promoted apoptosis, reduced Ki67 expression and enhanced cleaved caspase-3 expression in AML and ADR-resistant AML cells. In addition, CRNDE knockdown led to down-regulation of P-gp/MDR1, β-catenin, c-Myc and cyclinD1 expression, and enhanced the drug sensitivity to ADR in ADR-resistant AML cells. In conclusion, knockdown of CRNDE suppresses proliferation and P-gp-mediated MDR in ADR-resistant AML cells via inhibiting the Wnt/β-catenin pathway, suggesting that repression of CRNDE might be a therapeutic target to reverse MDR of ADR-resistant AML cells.

## Introduction

Acute myeloid leukemia (AML) is an aggressive hematopoietic system malignant disease, characterized by the clonal proliferation of myeloid precursors. AML still remains challenging to treat owing to patient factors (age and coexisting diseases) and intrinsic biologic factors [1]. Although advancement in survival rates by employing some targeted agents such as midostaurin and enasidenib, relapse rates remain high and most patients still die from AML [1,2]. Presently, drug resistance is a chief handicap to AML chemotherapy. In majority of AML patients who respond to the initial chemotherapy later still undergo multidrug resistance (MDR) and relapse have an adverse impact on patient survival [3]. Hence, there is a high medical need to formulate the possible molecular mechanism involved in MDR, improving the outcome of AML patients.

Long non-coding RNAs (lncRNAs) are RNAs that lack coding potential and are a heterogeneous class of transcripts longer than 200 nucleotides [4]. Growing data have indicated that abnormal regulation of lncRNAs is involved in various types of diseases, including cancer [5,6]. Dysregulation of lncRNAs has been reported to be involved in manifold cellular processes including cell proliferation, apoptosis, invasion, migration, and metastasis in many cancers [7]. Increasing evidence has shown that lncRNAs are linked to drug resistance or sensitivity and plenty of researches are concentrating on uncovering the definite molecular mechanism of lncRNA-modulated drug resistance [7]. Many lncRNAs have been reported to be involved in chemotherapy resistance of cancer cells, including hepatocellular carcinoma cells [8], human lung adenocarcinoma cells [9] and AML cells [10]. LncRNA CRNDE (CRNDE) is a gene locus hCG_1815491 on chromosome 16 located on the strand opposite to the adjacent *IRX5* gene and first confirmed to be elevated in colorectal adenomas [11]. The up-regulated CRNDE has been confirmed as a biomarker for AML and contributes to the malignant progression in AML cell line U937 [12]. However, the roles and specific mechanism of CRNDE in MDR in AML remains completely unclear.

In the present investigation, we explored the expression of CRNDE in peripheral blood monouclear cells (PBMCs) of patients with AML and after adriamycin (ADR)-based chemotherapy and parental as well as ADR-resistant AML cells. Further, we investigated the association between CRNDE expression and MDR-associated protein, multidrug resistance protein 1 (MDR1), and assessed the effects and potential mechanisms of CRNDE on proliferation and chemoresistance of AML cells. The results in the present study may contribute to a novel therapeutic strategy for reversing MDR in patients with AML.

## Materials and methods

### Patients and specimens

Twenty-nine patients who were diagnosed with AML at the First Affiliated Hospital of Zhengzhou University and ten healthy volunteers were enrolled into the present study. PBMC samples were collected from the AML patients before and after ADR-based chemotherapy between May 2016 and June 2017. All samples were enriched for mononuclear cells were purified from peripheral blood samples by standard Ficoll–Hypaque density gradient centrifugation from 10 to 40 ml PBS according to the reported methods [13] and then stored at −80°C until use. The study was approved by the Ethics Committee of the First Affiliated Hospital of Zhengzhou University. Written informed consent was obtained from the parent or legal guardian in accordance with the Helsinki Declaration.

### Cell culture

The human AML cell lines HL60, Kasumi-1, ADR-resistance HL60/ADR and human bone marrow stromal HS-5 cells were purchased from the Chinese Academy of Sciences (Shanghai, China). All cells were maintained by Dulbecco’s modified Eagle’s medium (DMEM; Life Technologies, Darmstadt, Germany) supplemented with 10% heat-inactivated fetal bovine serum (FBS; Gibco, Waltham, MA), 100 U/ml penicillin and 100 U/ml streptomycin in a humidified atmosphere of 5% CO_2_ at 37°C. To gain ADR-resistance Kasumi-1/ADR, ADR (Sigma, St. Louis, MO, U.S.A.) was used to treat Kasumi-1 cells at increasing concentrations of 0.2, 0.4, 0.8 and 1.6 μM. The HL60/ADR and Kasumi-1/ADR cells were cultured in the presence of 0.8 μM ADR. Before the experiment, ADR was withdrawn from the cells for 2 weeks. The cells were cultured in ADR-free medium for 2 weeks for further experiments before experiments.

### Lentivirus infection

Lentiviruses expressing short hairpin (sh) RNA targeting CRNDE, Wnt5a, and their corresponding controls were purchased from Gene Pharma (Shanghai, China), named sh-CRNDE, Lv-Wnt5a, Lv-Ctrl and sh-Ctrl, respectively. To obtain stable cell lines, 2 × 10^4^ HL60 and HL60/ADR cells were plated in 24-well plates, and after 24 h, the cells were infected with the recombinant lentiviruses (200 μl of viral supernatant) at 37°C for 2 days.

### RNA extraction and real-time quantitative RT-PCR

Total RNA was extracted from PBMCs, HL60, Kasumi-1, HL60/ADR, HS-5, HL60 transfected with sh-CRNDE and HL60/ADR transfected with sh-CRNDE cells. Total RNA extraction was performed using TRIzol reagent (Invitrogen, Karlsruhe, Germany) according to the manufacturer’s instructions. Then, reverse-transcribed into cDNA using the reverse transcription kit from Applied Biosystems (Foster City, CA, U.S.A.). The obtained cDNA was amplified in an ABI Prism 7900HT Fast Real-Time PCR System. mRNA relative expression levels of each gene were represented by 2^−ΔΔ*C*_t_^ method, and β-action was used as the internal reference. The primers used for qRT-PCR were as follows: CRNDE sense, 5′-TGA AGG AAG GAA GTG GTG CA-3′ and antisense, 5′-TCC AGT GGC ATC CTA CAA GA-3′; MDR1 sense, 5′-CCC ATC ATT GCA ATA GCAGG-3′ and antisense, 5′-GTT CAA ACT TCT GCT CCT GA-3′; β-actin: sense, 5′-GAC TTA GTT GCG TTA CAC CCT TTC-3′ and antisense, 5′-TGC TGT CAC CTT CAC CGT TC-3′.

For semi-quantitative PCR analyses, specific DNA fragments of CRNDE and β-actin were visualized by agarose gel electrophoresis.

### Western blot analysis

Cells were lysed in RIPA lysis buffer with proteinase inhibitor cocktail (Boster, Wuhan, China) and total proteins were extracted. Proteins were quantified using the BCA protein assay (Pierce, Rockford, IL, U.S.A.). Forty micrograms of proteins were separated by 10% SDS/PAGE, transferred on to PVDF membranes (Millipore, Billerica, MA, U.S.A.) and immune blotted with the respective primary antibodies at 4°C overnight. After washing three times, the membranes were incubated with HRP-linked secondary antibody at room temperature for 1 h. Protein bands were signaled using a chemiluminescence (ECL) reagent (Beyotime Biotechnology). The following antibodies were used in the present study with the respective concentration: p-glycoprotein (P-gp) (1:1000; Cell Signaling Technology, Danvers, MA, U.S.A.), Ki67 (1:200; Santa Cruz Biotechnology, Santa Cruz, CA), cleaved caspase-3 (1:1000; Cell Signaling Technology), β-catenin (1:400; Santa Cruz Biotechnology), c-Myc (1:1000; Cell Signaling Technology), cyclinD1 (1:800; Santa Cruz Biotechnology), β-actin (1:1000; Cell Signaling Technology), and IgG-horseradish peroxidase (HRP) (1:4000; Santa Cruz Biotechnology).

### Methyl-thiazol tetrazolium assays

Cell proliferation was determined using the methyl-thiazol tetrazolium (MTT) assays. The infected cells were plated at a density of 1 × 10^3^ cells/well into 96-well plates and cultured for the indicated times (0, 1, 2, 3 days). Then, 20 μl of MTT (Sigma–Aldrich, St. Louis, MO, U.S.A.) was added into each well and incubated for 4 h. Subsequently, 200 μl of DMSO (Tianjin Beilian Reagent Co., Ltd., China) was added into all wells to dissolve the formazan crystals. The determination of optical density (OD) values was conducted at 490 nm at different time points using a microplate reader absorbance test plate (Molecular Devices, Sunnyvale, CA, U.S.A.).

### Ethynyl-2′-deoxyuridine assays

Ethynyl-2′-deoxyuridine (EdU) assays were performed with EdU assay kit (Life Technologies Corporation, Carlsbad, CA, U.S.A.). Cells were cultured in 24-well plates with fresh laminin (Sigma–Aldrich, St. Louis, MO, U.S.A.) and incubated with EdU (final concentration, 10 μM) and Hoechst 33342 (5 μM) for 2 h according to the manufacturer’s instructions. Then the cells were fixed in 4% paraformaldehyde for 30 min at room temperature in the dark. Finally, the cells were observed and captured under an Olympus BX-51 microscope (Olympus, Tokyo, Japan). The number of EdU-positive cells was counted. The EdU incorporation rate was expressed as the ratio of EdU positive cells (green cells) to total Hoechst positive cells (blue cells).

### Flow cytometry assays

Cells were cultured for 48 h, and then were harvested, washed, and resuspended in pre-cold PBS. Apoptotic cells were identified by staining with Annexin V-PE/7-AAD (BD Pharmingen, Franklin Lakes, NJ, U.S.A.) according to the manufacturer’s instructions. The stained cells were analyzed by fluorescence activated cell sorting (FACS) (Beckman Coulter, Brea, CA, U.S.A.).

### Cytotoxicity assays

After 48 h of ADR drug treatment, cell viability of the infected cells was detected by MTT assays.

### Statistical analysis

All data were presented as mean plus standard deviation (SD). Statistical analyses were conducted using SPSS 21. Statistical differences between two groups were calculated by the Student’s *t* test. Pearson correlation coefficients were used to detect the relationship between CRNDE and MDR1 expression. *P*-value of <0.05 was considered as significant.

## Results

### CRNDE expression was up-regulated and positively associated with MDR1 expression in AML patients

**Figure 1:**
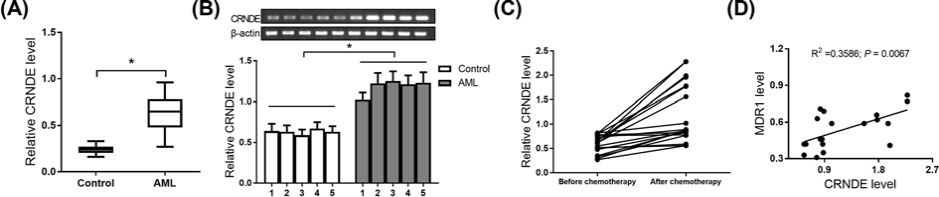
CRNDE expression was up-regulated and positively associated with MDR1 expression in 29 AML patients (**A**) The expression of CRNDE in AML patients was detected by qRT-PCR. (**B**) CRNDE expression in five cases of the randomly selected AML patients was measured by semi-quantitative analyses. (**C**) The expression of CRNDE in 19 AML patients before and after ADR-based chemotherapy was determined by qRT-PCR. (**D**) MDR1 level in 19 AML patients after ADR-based chemotherapy was detected by qRT-PCR. Pearson correlation coefficient was used to detect the relationship between CRNDE and MDR1 expression. *P<0.05.

The expression of CRNDE in PBMCs from AML patients was measured by qRT-PCR. As shown in Figure 1A, a remarkable increase in CRNDE was observed in AML patients compared with healthy control. Moreover, the results from semi-quantitative analyses disclosed that CRNDE expression was also significantly higher in five cases of the randomly selected AML patients than that in healthy control (Figure 1B). To determine whether expression of CRNDE is associated with AML drug resistance, the CRNDE and MDR1 expression was further detected by quantitative real-time polymerase chain reaction (qRT-PCR) in AML patients before and after ADR-based chemotherapy. qRT-PCR analyses showed that CRNDE level in AML patients after ADR-based chemotherapy was significantly up-regulated (Figure 1C). Furthermore, a significant positive correlation between the CRNDE level and MDR1 level was observed in AML patients after ADR-based chemotherapy (Figure 1D). The results indicated that CRNDE might affect drug resistance of AML patients.

### Expression profiles of CRNDE and MDR1 in AML cell lines

**Figure 2:**
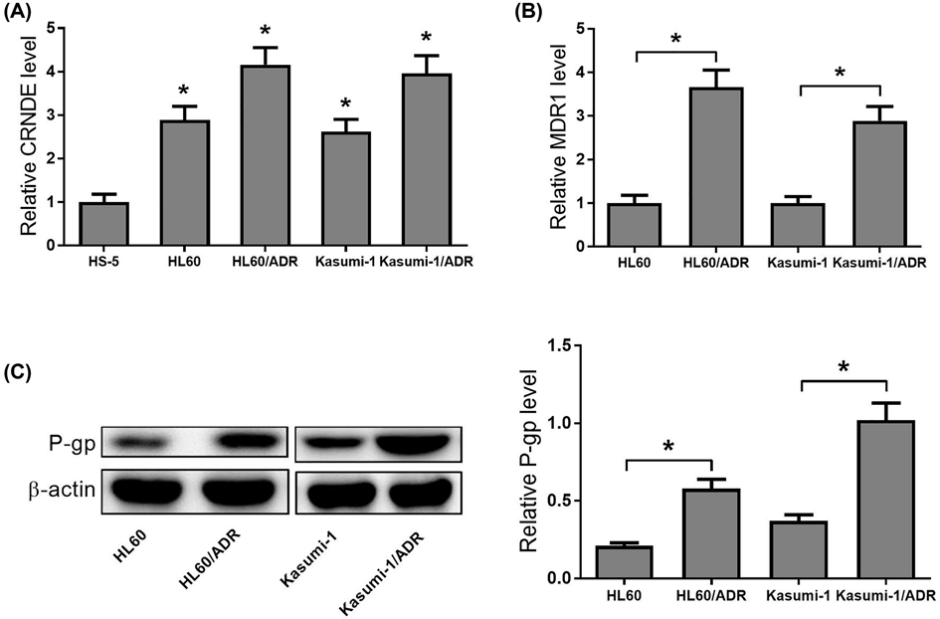
Expression profiles of CRNDE and MDR1 in AML cell lines (**A**) qRT-PCR was used to detect the CRNDE expression in two ADR-resistant cell lines (HL60/ADR and Kasumi-1/ADR cells) and their parent cell lines. (**B,C**) qRT-PCR and Western blot assays were used to measure the MDR1/P-gp expression levels in two ADR-resistant cell lines (HL60/ADR and Kasumi-1/ADR cells) and their parent cell lines. *P<0.05.

The levels of CRNDE in two ADR-resistant cell lines (HL60/ADR and Kasumi-1/ADR cells) compared with parent cell lines (HL60 and Kasumi-1 cells) were analyzed by qRT-PCR. Results from qRT-PCR analyses showed that CRNDE expression was significantly up-regulated in two ADR-resistant cell lines compared with drug-susceptible parental cell lines (Figure 2A). In addition, MDR1/P-gp expression in two ADR-resistant cell lines was determined by qRT-PCR and Western blot analyses. As shown in Figure 2B,C, MDR1/P-gp expression was significantly increased in two ADR-resistant cell lines compared with parental cell lines. These data further suggested that CRNDE might participate in drug resistance of AML.

### Knockdown of CRNDE inhibited proliferation and promoted apoptosis in AML cell lines

**Figure 3:**
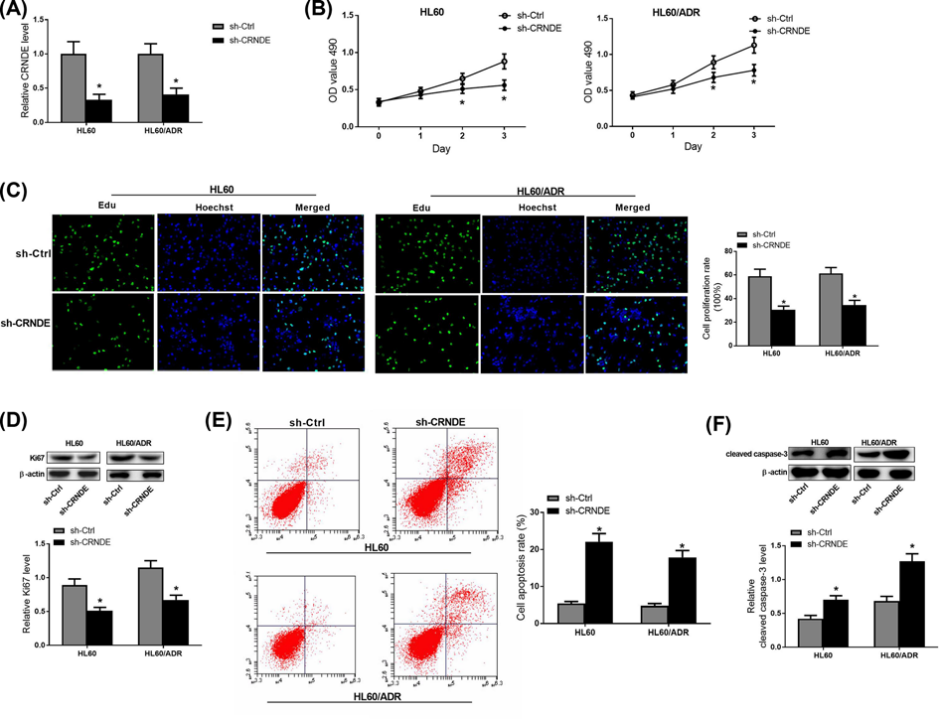
Knockdown of CRNDE inhibited proliferation and promoted apoptosis in AML cell lines (**A**) CRNDE expression was detected by qRT-PCR in HL60/ADR and parent cell lines transfected with sh-CRNDE. (**B**) Cell proliferation of the transfected HL60/ADR and parent cells was detected by MTT assays. (**C**) EdU assays were conducted to assess cell proliferation of the transfected HL60/ADR and parent cells. (**D**) Western blot was used to detect expression of Ki67 in the transfected HL60/ADR and parent cells. (**E**) The apoptosis in the transfected HL60/ADR and parent cells was detected by flow cytometry assays. (**F**) Western blot was used to detect expression of cleaved caspase-3 in the transfected HL60/ADR and parent cells. *P<0.05.

To explore the roles of CRNDE in AML cells, CRNDE was knocked down in HL60 and HL60/ADR cells and some functional experiments were performed. As shown Figure 3A, CRNDE level was significantly reduced in sh-CRNDE transfected HL60 and HL60/ADR cells compared with their corresponding controls. Then, MTT and EdU assays were performed to detect the proliferative capabilities of AML cell lines. These results revealed that a reduced capability to proliferate in sh-CRNDE transfected HL60 and HL60/ADR cells compared with their control groups (Figure 3B,C). Moreover, Western blot analyses showed that knockdown of CRNDE significantly suppressed the expression of proliferation-related protein Ki67 (Figure 3D). In addition, flow cytometry assays were conducted to detect the effect of knockdown of CRNDE on apoptosis in HL60 and HL60/ADR cells. Apoptosis rates in HL60 and HL60/ADR cells transfected with sh-CRNDE were significantly increased compared with their controls (Figure 3E). Furthermore, Western blot analyses showed that the expression of cleavage caspase-3 was obviously up-regulated in sh-CRNDE transfected HL60 and HL60/ADR cells (Figure 3F). These results suggested that knockdown of CRNDE inhibited proliferation and promoted apoptosis in AML and ADR-resistant AML cells.

### Knockdown of CRNDE enhanced the chemosensitivity of AML cells *in vitro*

**Figure 4:**
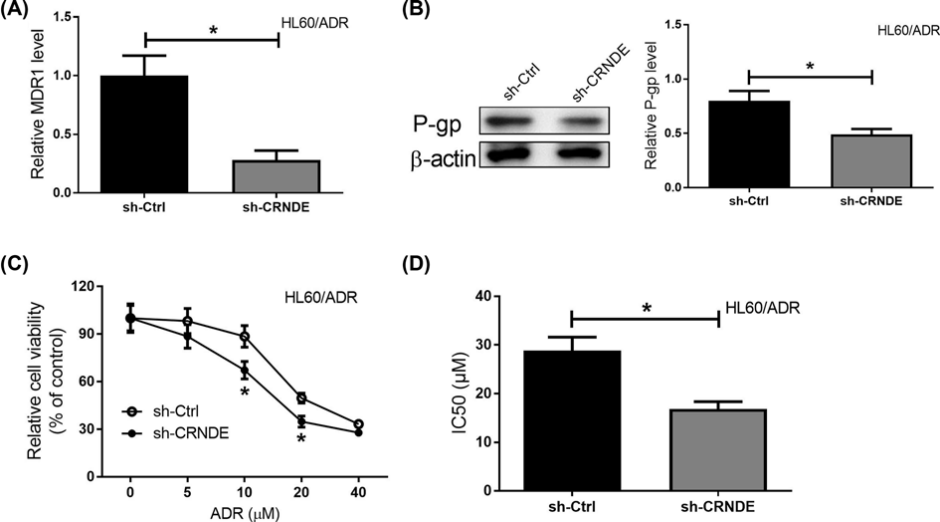
Knockdown of CRNDE promoted the chemosensitivity of AML cells *in vitro* (**A,B**) MDR1/P-gp expression levels in HL60/ADR cells transfected with sh-CRNDE. (**C**) Cell viability of HL60/ADR cells transfected with sh-CRNDE in response to different concentrations of ADR was detected by MTT assays. (**D**) The IC_50_ values of cells were calculated. *P<0.05.

To investigate the effects of CRNDE on chemosensitivity in AML cells, CRNDE was knocked down in HL60/ADR cells. Further, the MDR1/P-gp expression was measured by qRT-PCR and Western blot analyses and the results showed that CRNDE knockdown remarkably repressed MDR1/P-gp expression in HL60/ADR cells (Figure 4A,B). To further assess the sensitivity to ADR in HL60/ADR cells, cytotoxicity assays were performed. Interestingly, CRNDE knockdown enhanced the sensitivity to ADR in HL60/ADR cells (Figure 4C). Moreover, the IC_50_ values in HL60/ADR cells transfected with sh-CRNDE were obviously reduced compared with sh-Ctr group (Figure 4D). These results showed that CRNDE knockdown constrained drug resistance in AML cells.

### Knockdown of CRNDE promoted the chemosensitivity of AML cells through inactivating the Wnt/β-catenin pathway

**Figure 5:**
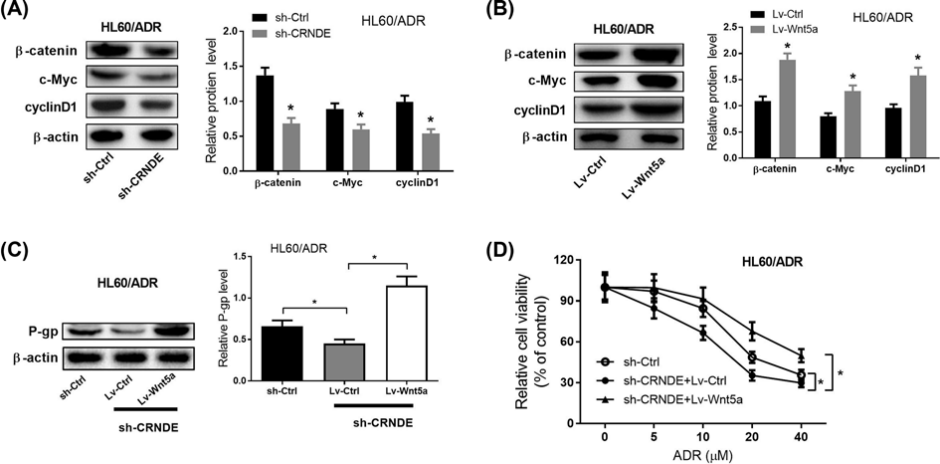
Knockdown of CRNDE promoted the chemosensitivity of AML cells through constraining the Wnt/β-catenin pathway (**A**) Western blot was used to detect the expression of β-catenin, c-Myc and cyclinD1 in HL60/ADR cells transfected with sh-CRNDE. (**B**) The expression of β-catenin, c-Myc and cyclinD1 in HL60/ADR cells transfected with Lv-Wnt5a. (**C**) P-gp expression in HL60/ADR transfected with sh-CRNDE or/and Lv-Wnt5a. (**D**) Cell viability of HL60/ADR cells transfected with sh-CRNDE or/and Lv-Wnt5a in response to different concentrations of ADR was detected by MTT assays. *P<0.05.

A previous study has reported that inhibition of the Wnt/β-catenin signaling induced by CRNDE knockdown can reduce the chemoresistance of colorectal cancer (CRC) cells [14]. To investigate whether CRNDE affects chemosensitivity of AML cells, Western blot assays were conducted to detect the expression of intracellular signal transducer β-catenin and its downstream target genes (*c-Myc* and *cyclinD1*) in the Wnt/β-catenin pathway in HL60/ADR cells. Results from Western blot analyses showed that CRNDE down-regulation obviously repressed the expression of β-catenin, c-Myc, and cyclinD1 in HL60/ADR cells (Figure 5A). Moreover, Wnt5a overexpression (Wnt5a can activate the Wnt/β-catenin signaling) significantly promoted the expression of β-catenin, c-Myc, and cyclinD1 in HL60/ADR cells (Figure 5B). Interestingly, Wnt5a overexpression inversed the inhibitory effect of CRNDE knockdown on P-gp expression in HL60/ADR cells (Figure 5C). Furthermore, Wnt5a overexpression inversed the positive effect of CRNDE knockdown on the sensitivity to different concentrations of ADR in HL60/ADR cells (Figure 5D). These results concluded that CRNDE knockdown enhanced the chemosensitivity of AML cells through repressing the Wnt/β-catenin pathway.

## Discussion

AML is a hematopoietic malignancy with large heterogeneity both in cell genetics and molecular genetics, and chemoresistance resulting from frequent secondary AML tends to result in poor prognosis of patients with AML [3]. Clinically, MDR remains one of the main obstacles to gain successful tumor chemotherapy. Some studies have revealed that mechanisms of MDR include alteration of the cell environment, suppression of apoptosis and drug efflux mediated by ABC transporters [15]. In our present study, we focused on exploring the expression of CRNDE in AML patients and investigating the association between CRNDE expression and MDR-associated protein, MDR1, and the effects and potential mechanisms of CRNDE on proliferation and chemoresistance of AML cells.

With the accumulating understanding of the lncRNA function, increasing evidence has indicated that lncRNAs are involved in multiple cellular processes such as proliferation, migration, invasion, apoptosis and chemoresistance in cancers [16–18]. CRNDE was first confirmed as a functional lncRNA gene in CRC [19]. Increasing evidence has shown that CRNDE is involved in modulating cancer cell proliferation, migration, invasion, apoptosis, and chemoresistance [20–22]. For example, in CRC, CRNDE has been reported to modulate progression and chemoresistance of CRC through regulating miR-181a-5p expression and activating the Wnt/β-catenin signaling [14]. A previous study has shown that CRNDE is highly expressed in gastric cancer (GC) cell lines and tissues and CRNDE overexpression promotes cell proliferation [23]. Furthermore, lncRNAs, including CRNDE, have been reported to function in AML pathogenesis [12,24]. In our present study, up-regulation of CRNDE and a positive correlation between the levels of CRNDE and MDR1 were observed in patients with AML, especially in AML patients after ADR-based chemotherapy, which revealed that CRNDE was involved in the development of MDR in AML. To explore the association between CRNDE and chemoresistance *in vitro*, loss of CRNDE function assays were performed in ADR-resistant AML cells. Our results showed that CRNDE down-regulation inhibited cell proliferation and promoted apoptosis in ADR-resistant AML cells, which was consistent with a previous research [12].

Involved in multiple cancer-related signaling pathways, CRNDE plays important roles in development of cancers [25–27]. Some evidence has revealed that CRNDE can modulate the progression and chemoresistance of cancers by regulating the Wnt/β-catenin signaling [14,28]. Mounting studies have exhibited that the Wnt/β-catenin signaling is involved in cell proliferation and chemoresistance. The Wnt/β-catenin pathway regulates expression and function of many proteins that are necessary for leukemia cell MDR [29]. Moreover, some evidence has shown that the maintaining of MDR in cancer cells by the Wnt/β-catenin pathway is significantly related to P-gp [30,31]. Notably, our results showed that CRNDE knockdown led to down-regulation of P-gp/MDR1 and enhanced the drug sensitivity to ADR in AML and ADR-resistant AML cells. The specific downstream genes of Wnt/β-catenin pathway (c-Myc and cyclinD1) have been reported to play a crucial role in cell functions [32,33]. Therefore, to further explore the potential molecular mechanism, we detected the expression of P-gp/MDR1, β-catenin, c-Myc and cyclinD1 in treated ADR-resistant AML cells. We found that CRNDE down-regulation enhanced the sensitivity to ADR in ADR-resistant AML cells through inactivating the Wnt/β-catenin pathway.

In conclusion our findings concluded that CRNDE down-regulation inhibited cell proliferation and P-gp-mediated MDR in AML via repressing the Wnt/β-catenin pathway, suggesting that repression of CRNDE might be a therapeutic target to reverse MDR of ADR-resistant AML cells. The underlying mechanisms of chemotherapy resistance will help to find novel targets to reverse MDR in AML.

## Abbreviations

ADR: adriamycin
AML: acute myeloid leukemia
CRC: colorectal cancer
CRNDE: colorectal neoplasia differentially expressed
EdU: ethynyl-2′-deoxyuridine
HRP: horseradish peroxidase
lncRNA: long non-coding RNA
MDR: multidrug resistance
MDR1: multidrug resistance protein 1
MTT: methyl-thiazol tetrazolium
OD: optical density
PBMC: peripheral blood monouclear cell
PE: phycoerythrin
P-gp: p-glycoprotein
qRT-PCR: quantitative real-time polymerase chain reaction
7-AAD: 7-amino-actinomycin D 7

## References

[1] Stone R.M., Mandrekar S.J., Sanford B.L., Laumann K., Geyer S., Bloomfield C.D. et al. (2017) Midostaurin plus chemotherapy for acute myeloid leukemia with a FLT3 mutation. N. Engl. J. Med. 377, 454–464 10.1056/NEJMoa1614359 28644114 PMC5754190

[2] (2017) Positive first trial of enasidenib for AML. Cancer Discov. 7, 10.1158/2159-8290.CD-NB2017-098 28659444

[3] Briot T., Roger E., Thepot S. and Lagarce F. (2018) Advances in treatment formulations for acute myeloid leukemia. Drug Discov. Today 23, 1936–1949 10.1016/j.drudis.2018.05.040 29870791

[4] Wang P., Chen D., Ma H. and Li Y. (2017) LncRNA SNHG12 contributes to multidrug resistance through activating the MAPK/Slug pathway by sponging miR-181a in non-small cell lung cancer. Oncotarget 8, 84086–84101 10.18632/oncotarget.20475 29137407 PMC5663579

[5] Wang B.G., Lv Z., Ding H.X., Fang X.X., Wen J., Xu Q. et al. (2018) The association of lncRNA-HULC polymorphisms with hepatocellular cancer risk and prognosis. Gene 670, 148–154 10.1016/j.gene.2018.05.096 29803923

[6] Wang Y., Wu K., Yang Z., Zhao Q., Fan D., Xu P. et al. (2015) Multidrug-resistance related long non-coding RNA expression profile analysis of gastric cancer. PLoS ONE 10, e0135461 10.1371/journal.pone.0135461 26291830 PMC4546299

[7] Chen Q.N., Wei C.C., Wang Z.X. and Sun M. (2017) Long non-coding RNAs in anti-cancer drug resistance. Oncotarget 8, 1925–1936 10.18632/oncotarget.12461 27713133 PMC5352108

[8] Yuan P., Cao W., Zang Q., Li G., Guo X. and Fan J. (2016) The HIF-2alpha-MALAT1-miR-216b axis regulates multi-drug resistance of hepatocellular carcinoma cells via modulating autophagy. Biochem. Biophys. Res. Commun. 478, 1067–1073 10.1016/j.bbrc.2016.08.065 27524242

[9] Liu Z., Sun M., Lu K., Liu J., Zhang M., Wu W. et al. (2013) The long noncoding RNA HOTAIR contributes to cisplatin resistance of human lung adenocarcinoma cells via downregualtion of p21(WAF1/CIP1) expression. PLoS ONE 8, e77293 10.1371/journal.pone.0077293 24155936 PMC3796503

[10] Liu B., Ma X., Liu Q., Xiao Y., Pan S. and Jia L. (2018) Aberrant mannosylation profile and FTX/miR-342/ALG3-axis contribute to development of drug resistance in acute myeloid leukemia. Cell Death Dis. 9, 688 29880818 10.1038/s41419-018-0706-7 PMC5992136

[11] Zhang J., Yin M., Peng G. and Zhao Y. (2018) CRNDE: an important oncogenic long non-coding RNA in human cancers. Cell Prolif. 51, e12440 10.1111/cpr.12440 29405523 PMC6528921

[12] Wang Y., Zhou Q. and Ma J.J. (2018) High expression of lnc-CRNDE presents as a biomarker for acute myeloid leukemia and promotes the malignant progression in acute myeloid leukemia cell line U937. Eur. Rev. Med. Pharmacol. Sci. 22, 763–770 29461608 10.26355/eurrev_201802_14310

[13] Corkum C.P., Ings D.P., Burgess C., Karwowska S., Kroll W. and Michalak T.I. (2015) Immune cell subsets and their gene expression profiles from human PBMC isolated by Vacutainer Cell Preparation Tube (CPT) and standard density gradient. BMC Immunol. 16, 48 10.1186/s12865-015-0113-0 26307036 PMC4549105

[14] Han P., Li J.W., Zhang B.M., Lv J.C., Li Y.M., Gu X.Y. et al. (2017) The lncRNA CRNDE promotes colorectal cancer cell proliferation and chemoresistance via miR-181a-5p-mediated regulation of Wnt/beta-catenin signaling. Mol. Cancer 16, 9 10.1186/s12943-017-0583-1 28086904 PMC5237133

[15] Ma H., Cheng L., Hao K., Li Y., Song X., Zhou H. et al. (2014) Reversal effect of ST6GAL 1 on multidrug resistance in human leukemia by regulating the PI3K/Akt pathway and the expression of P-gp and MRP1. PLoS ONE 9, e85113 10.1371/journal.pone.0085113 24454800 PMC3894187

[16] Bartonicek N., Maag J.L. and Dinger M.E. (2016) Long noncoding RNAs in cancer: mechanisms of action and technological advancements. Mol. Cancer 15, 43 10.1186/s12943-016-0530-6 27233618 PMC4884374

[17] Lavorgna G., Vago R., Sarmini M., Montorsi F., Salonia A. and Bellone M. (2016) Long non-coding RNAs as novel therapeutic targets in cancer. Pharmacol. Res. 110, 131–138 10.1016/j.phrs.2016.05.018 27210721

[18] Qi P., Zhou X.Y. and Du X. (2016) Circulating long non-coding RNAs in cancer: current status and future perspectives. Mol. Cancer 15, 39 10.1186/s12943-016-0524-4 27189224 PMC4869386

[19] Graham L.D., Pedersen S.K., Brown G.S., Ho T., Kassir Z., Moynihan A.T. et al. (2011) Colorectal neoplasia differentially expressed (CRNDE), a novel gene with elevated expression in colorectal adenomas and adenocarcinomas. Genes Cancer 2, 829–840 10.1177/1947601911431081 22393467 PMC3278902

[20] Yu B., Ye X., Du Q., Zhu B., Zhai Q. and Li X.X. (2017) The long non-coding RNA CRNDE promotes colorectal carcinoma progression by competitively binding miR-217 with TCF7L2 and enhancing the Wnt/beta-catenin signaling pathway. Cell. Physiol. Biochem. 41, 2489–2502 10.1159/000475941 28472810

[21] Szafron L.M., Balcerak A., Grzybowska E.A., Pienkowska-Grela B., Podgorska A., Zub R. et al. (2015) The putative oncogene, CRNDE, is a negative prognostic factor in ovarian cancer patients. Oncotarget 6, 43897–43910 10.18632/oncotarget.6016 26556866 PMC4791275

[22] Kiang K.M., Zhang X.Q., Zhang G.P., Li N., Cheng S.Y., Poon M.W. et al. (2017) CRNDE expression positively correlates with EGFR activation and modulates glioma cell growth. Target Oncol. 12, 353–363 28493025 10.1007/s11523-017-0488-3

[23] Hu C.E., Du P.Z., Zhang H.D. and Huang G.J. (2017) Long noncoding RNA CRNDE promotes proliferation of gastric cancer cells by targeting miR-145. Cell. Physiol. Biochem. 42, 13–21 10.1159/000477107 28490034

[24] Kung J.T., Colognori D. and Lee J.T. (2013) Long noncoding RNAs: past, present, and future. Genetics 193, 651–669 10.1534/genetics.112.146704 23463798 PMC3583990

[25] Zheng J., Liu X., Wang P., Xue Y., Ma J., Qu C. et al. (2016) CRNDE promotes malignant progression of glioma by attenuating miR-384/PIWIL4/STAT3 Axis. Mol. Ther. 24, 1199–1215 10.1038/mt.2016.71 27058823 PMC5088760

[26] Ellis B.C., Graham L.D. and Molloy P.L. (2014) CRNDE, a long non-coding RNA responsive to insulin/IGF signaling, regulates genes involved in central metabolism. Biochim. Biophys. Acta 1843, 372–386 10.1016/j.bbamcr.2013.10.016 24184209

[27] Du D.X., Lian D.B., Amin B.H. and Yan W. (2017) Long non-coding RNA CRNDE is a novel tumor promoter by modulating PI3K/AKT signal pathways in human gastric cancer. Eur. Rev. Med. Pharmacol. Sci. 21, 5392–5398 29243780 10.26355/eurrev_201712_13925

[28] Hu K., Gu Y., Lou L., Liu L., Hu Y., Wang B. et al. (2015) Galectin-3 mediates bone marrow microenvironment-induced drug resistance in acute leukemia cells via Wnt/beta-catenin signaling pathway. J. Hematol. Oncol. 8, 1 10.1186/s13045-014-0099-8 25622682 PMC4332970

[29] Hamdoun S., Fleischer E., Klinger A. and Efferth T. (2017) Lawsone derivatives target the Wnt/beta-catenin signaling pathway in multidrug-resistant acute lymphoblastic leukemia cells. Biochem. Pharmacol. 146, 63–73 10.1016/j.bcp.2017.10.008 29061340

[30] Xia Z., Guo M., Liu H., Jiang L., Li Q., Peng J. et al. (2015) CBP-dependent Wnt/beta-catenin signaling is crucial in regulation of MDR1 transcription. Curr. Cancer Drug Targets 15, 519–532 10.2174/1568009615666150506093643 25968898

[31] Wang B., Zou Q., Sun M., Chen J., Wang T., Bai Y. et al. (2014) Reversion of trichostatin A resistance via inhibition of the Wnt signaling pathway in human pancreatic cancer cells. Oncol. Rep. 32, 2015–2022 10.3892/or.2014.3476 25224651

[32] Shtutman M., Zhurinsky J., Simcha I., Albanese C., D’Amico M., Pestell R. et al. (1999) The cyclin D1 gene is a target of the beta-catenin/LEF-1 pathway. Proc. Natl. Acad. Sci. U.S.A. 96, 5522–5527 10.1073/pnas.96.10.5522 10318916 PMC21892

[33] He T.C., Sparks A.B., Rago C., Hermeking H., Zawel L., da Costa L.T. et al. (1998) Identification of c-MYC as a target of the APC pathway. Science 281, 1509–1512 10.1126/science.281.5382.1509 9727977

